# Analysis of risk factors associated with pulmonary embolism in patients with spinal cord injury: A meta-analysis

**DOI:** 10.1016/j.clinsp.2026.101095

**Published:** 2026-09-06

**Authors:** Cheng-Qun Sun, Ke-Yong Liang

**Affiliations:** Department of Spine Surgery, The Affiliated Yangming Hospital of Ningbo University, Yuyao, Zhejiang, China

**Keywords:** Pulmonary embolism, Spinal cord injury, Risk factors, Meta-analysis, Venous thromboembolism

## Abstract

**Background:**

Pulmonary Embolism (PE) represents a life-threatening complication in patients with Spinal Cord Injury (SCI), yet comprehensive evidence regarding its risk factors remains limited. This meta-analysis aimed to systematically evaluate risk factors associated with PE development in SCI patients.

**Methods:**

A systematic literature search was conducted across major electronic databases (PubMed, Cochrane database, Embase, CNKI, and Wanfang), identifying studies that reported risk factors for PE in SCI patients. Pooled Odds Ratios (ORs) with 95% Confidence Intervals (95% CIs) were calculated using fixed-effect or random-effects models. Study quality was evaluated using the Newcastle-Ottawa Scale.

**Results:**

Nine studies encompassing 394,322 SCI patients were included, with an overall PE incidence of 0.125%. Meta-analysis revealed that older age (OR = 1.80, 95% CI 1.49–2.17, p < 0.001), prolonged operative time (OR = 2.56, 95% CI 1.84–3.56, p < 0.001), history of deep vein thrombosis or PE (OR = 29.10, 95% CI 14.03–60.37, p < 0.001), and traumatic injury etiology (OR = 3.15, 95% CI 1.37–7.22, p = 0.007) were significantly associated with increased PE risk. Conversely, sex, body mass index, and comorbidities showed no significant associations with PE development. Sensitivity analyses confirmed the robustness of these findings.

**Conclusions:**

This meta-analysis identifies older age, prolonged operative time, prior thromboembolic history, and traumatic injury as significant risk factors for PE in SCI patients. These findings provide evidence-based guidance for risk stratification and implementation of targeted prophylactic strategies in this vulnerable population.

## Highlights

• Identified key risk factors for PE in SCI patients.
• Prior DVT/PE showed strongest association (OR = 29.10).
• Findings support SCI-specific risk stratification.

## Introduction

Spinal Cord Injury (SCI) represents a catastrophic neurological condition with profound implications for individuals and healthcare systems worldwide. Global incidence estimates range from 10.4 to 83 cases per million population annually, exhibiting substantial geographic heterogeneity influenced by socioeconomic development, traffic safety infrastructure, and injury prevention policies.^1^ Contemporary systematic analyses report a pooled incidence of 23.77 per million, with traumatic mechanisms historically accounting for approximately 90% of cases.^2^ However, recent epidemiological shifts in developed nations reveal an evolving landscape, where non-traumatic SCI now constitutes up to 40% of cases ‒ a trend attributable to population aging and increasing prevalence of degenerative spinal pathology.^3^ Beyond immediate neurological sequelae, SCI patients confront a cascade of secondary complications that profoundly influence long-term morbidity, mortality, and functional independence. Among these multifaceted complications ‒ ranging from pressure ulceration and urinary sepsis to chronic pain and respiratory insufficiency ‒ Venous Thromboembolism (VTE) emerges as one of the most clinically consequential, particularly during acute and subacute recovery phases when Deep Vein Thrombosis (DVT) occurs in 10%‒100% of patients without prophylaxis.^4^^,^^5^

Pulmonary Embolism (PE) constitutes a life-threatening manifestation of VTE in SCI patients, with documented incidence rates of 0.6%‒5% during hospitalization ‒ markedly exceeding background population risk.^6^ Despite contemporary prophylactic strategies, PE persists as a leading cause of mortality, contributing to 9.7% of deaths within the first post-injury year.^7^ The clinical recognition of PE in this population presents formidable diagnostic challenges uniquely compounded by neurological deficits. Sensory impairment masks characteristic symptoms including pleuritic chest pain and lower extremity discomfort, while motor paralysis obscures physical signs of underlying deep vein thrombosis. Dyspnea may be erroneously attributed to baseline respiratory compromise from high cervical lesions, and presentations manifesting solely as unexplained fever or non-specific tachycardia further confound clinical assessment. Systematic screening protocols reveal that up to 70% of DVT cases remain clinically silent until advanced stages, substantially elevating progression risk to PE.^8^ These diagnostic complexities contribute to treatment delays and excess mortality. The ramifications of PE extend beyond immediate mortality risk to encompass disrupted rehabilitation trajectories, mandatory prolonged anticoagulation with inherent hemorrhagic complications, and substantially amplified healthcare resource utilization. Patients experiencing PE face extended hospitalizations, interrupted mobilization protocols, and attenuated functional recovery ‒ collectively compromising long-term quality of life and community reintegration.

The pathophysiological mechanisms underlying PE development in SCI patients reflect complex interactions encompassing all elements of Virchow's classical triad: venous stasis, vascular endothelial injury, and systemic hypercoagulability. Immobilization consequent to neurological impairment produces profound lower extremity venous stasis through elimination of skeletal muscle pump function and autonomic dysregulation affecting venous compliance. Neurogenic paralysis culminates in venous dilation, increased capacitance, and diminished flow velocity, establishing optimal conditions for thrombogenesis.^9^ The acute injury phase initiates a potent inflammatory cascade characterized by sequential macrophage activation, neutrophil infiltration, and pro-inflammatory cytokine elaboration, with peak activity occurring 7‒14 days post-injury ‒ temporally coincident with maximal DVT incidence.10., 11., 12. Key inflammatory mediators including tumor necrosis factor-alpha, interleukin-1β, and interleukin-6 induce endothelial dysfunction while upregulating tissue factor expression, thereby activating the extrinsic coagulation pathway.^13^ Endothelial injury arises from direct mechanical trauma, sustained inflammatory stress, and regional hypoxia secondary to venous stasis.^14^ The resultant hypercoagulable state manifests through elevated thrombin generation markers and increased circulating fibrinogen.^15^^,^^16^ Importantly, impaired fibrinolysis ‒ driven by markedly elevated Plasminogen Activator Inhibitor-1 (PAI-1) levels ‒ plays a dominant pathogenic role by compromising endogenous clot resolution mechanisms and promoting thrombus persistence.^14^ This prothrombotic milieu may persist for weeks to months post-injury, creating a sustained temporal window of heightened PE vulnerability.^14^^,^15., 16., 17.

Despite widespread recognition of PE as a major SCI complication, current risk assessment methodologies remain inadequately tailored to this population's unique characteristics. Widely employed VTE risk stratification instruments, including the Caprini score and Padua prediction score, were derived and validated in heterogeneous surgical and general medical cohorts. These tools universally categorize all SCI patients as “high risk” without enabling meaningful within-group risk differentiation.^18^^,^^19^ Critically, they fail to incorporate SCI-specific determinants such as neurological injury level, American Spinal Injury Association (ASIA) Impairment Scale grade, temporal proximity to injury, and concurrent traumatic injuries including lower extremity or pelvic fractures ‒ factors potentially modulating thrombotic risk. Recent validation efforts demonstrate suboptimal discriminative performance of generic tools when applied to trauma populations with SCI. Furthermore, existing clinical practice guidelines for VTE prophylaxis exhibit substantial heterogeneity across professional societies. Major areas of divergence include optimal anticoagulation initiation timing, prophylaxis duration, agent selection, and the incremental benefit of combined mechanical-pharmacological approaches. This guideline discordance reflects an insufficient evidence foundation and engenders considerable inter-institutional practice variation, ultimately limiting clinicians' capacity to implement individualized, risk-stratified prevention strategies.

Multiple single-center observational investigations have explored potential PE risk factors in SCI populations, examining associations with demographic variables (age, sex), anthropometric measures (Body Mass Index [BMI]), injury characteristics (severity, level, completeness), therapeutic interventions (surgical duration), and comorbidity profiles.20., 21., 22., 23. However, these studies have generated inconsistent and occasionally contradictory findings that preclude definitive conclusions. Illustratively, while several investigations identify advancing age as a significant predictor, the large California state-wide database analysis by Jones et al. encompassing 16,240 SCI patients did not detect an independent age-related association.^6^ Similarly, conflicting evidence characterizes the relationship between injury level and PE risk, with studies variably reporting elevated risk in paraplegia versus tetraplegia or finding no differential effect. Such inconsistencies likely reflect fundamental methodological limitations: small sample sizes constraining statistical power, single-center designs limiting generalizability, heterogeneous outcome ascertainment methods, incomplete adjustment for potential confounders, and variable diagnostic screening intensity. Consequently, the evidence base remains fragmented, effect magnitude estimates lack precision, and the relative prognostic importance of individual factors remains incompletely characterized ‒ substantially impeding development of robust, clinically actionable prediction models.

Current evidence regarding PE risk factors in SCI patients exhibits critical deficiencies that constrain clinical translation. The literature remains dispersed across numerous small studies yielding conflicting conclusions, effect size estimates are imprecise with wide confidence intervals reflecting substantial heterogeneity, and genuinely independent risk factors remain unestablished due to inadequate multivariable confounder adjustment. Importantly, existing meta-analyses have predominantly addressed generic VTE outcomes rather than PE-specific endpoints, despite distinct pathophysiological mechanisms, clinical sequelae, and mortality implications. To address these knowledge gaps, we conducted a comprehensive systematic review and meta-analysis specifically focused on identifying and quantifying risk factors associated with PE development in SCI patients. Through rigorous pooling of data across multiple studies, this analysis provides enhanced statistical power to detect true associations while generating precise effect magnitude estimates. Our primary objective was to identify clinically meaningful, independently predictive risk factors capable of informing evidence-based risk stratification frameworks and guiding implementation of targeted, individualized prophylactic interventions. These findings are intended to optimize preventive care delivery and ultimately reduce PE-related morbidity and mortality in this uniquely vulnerable patient population.

## Materials and methods

This systematic review and meta-analysis was conducted and reported in accordance with the Preferred Reporting Items for Systematic Reviews and Meta-Analyses (PRISMA) 2020 statement.^24^ The review protocol was registered on OSF platform (registration DOI: 10.17,605/OSF.IO/479GS: https://osf.io/vb6kg). The completed PRISMA 2020 Checklist is provided as Supplementary File S1.

### Search strategy

A comprehensive systematic literature search was conducted in PubMed (from 1966), the Cochrane Library (from 1996), Embase (from 1947), China National Knowledge Infrastructure (CNKI; from 1999), and Wanfang Database (from 1993), with all searches updated through 30 September 2025. The search strategy employed a combination of Medical Subject Headings (MeSH) terms and free-text words related to spinal cord injury and pulmonary embolism. The following search terms were used: “spinal cord injury” OR “spinal cord trauma” OR “SCI” OR “paraplegia” OR “tetraplegia” AND “pulmonary embolism” OR "PE" OR “pulmonary thromboembolism” OR “venous thromboembolism”. Languages were restricted to Chinese and English. Reference lists of all included studies and of prior relevant systematic reviews were manually screened to identify additional eligible studies.

### Inclusion and exclusion criteria

The inclusion criteria were as follows: 1) Study population consisted of patients diagnosed with SCI, regardless of age, injury level, or completeness of injury; 2) Cohort studies, case-control studies, or cross-sectional studies with appropriate comparison groups; 3) Multivariable analysis was performed. The exclusion criteria were: 1) Case reports, case series, narrative reviews, systematic reviews, meta-analyses, editorials, commentaries, letters, or conference abstracts; 2) Studies lacking multivariable analysis; 3) Duplicate publications or studies reporting on the same patient cohort; and 4) Animal studies or in vitro experimental studies.

### Data extraction and quality assessment

Data extraction was performed independently by two investigators using a standardized data collection form. The following information was extracted from each included study: first author, publication year, country, sample size, age, sex distribution, percentage of pulmonary embolism cases. The methodological quality of included studies was evaluated using the Newcastle-Ottawa Scale (NOS), which is specifically designed for assessing non-randomized studies in meta-analyses. The NOS employs a star-based rating system across three domains: selection of study groups (0‒4 stars), comparability of groups (0‒2 stars), and ascertainment of outcome or exposure (0‒3 stars). Studies scoring ≥ 7 stars were classified as high quality, 5‒6 stars as moderate quality, and < 5 stars as low quality. Quality assessment was performed independently by two reviewers, with disagreements resolved through consensus discussion.

### Statistical analysis

The meta-analysis was conducted in accordance with the methodological recommendations of the Cochrane Handbook for Systematic Reviews of Interventions, version 6.4.^25^ The adjusted Odds Ratios (ORs) with 95% Confidence Intervals (95% CIs) for each risk factor associated with PE in SCI patients were prioritized for extraction and subsequently log-transformed. Statistical heterogeneity among studies was assessed using the *I*^2^ statistic and Cochran's *Q*-test. *I*^2^ values of < 25%, 25%‒50%, 50%‒75%, and > 75% were considered to indicate low, moderate, substantial, and considerable heterogeneity, respectively. When heterogeneity was low to moderate (*I*^2^ ≤ 50% and p ≥ 0.10), a fixed-effect model using the Mantel-Haenszel method was employed. Conversely, when substantial or considerable heterogeneity was detected (*I*^2^ > 50% or p < 0.10), a random-effects model using the DerSimonian-Laird method was applied. Sensitivity analyses were performed by sequentially excluding individual studies to evaluate the stability and robustness of the pooled estimates. Meta-regression was not performed because, per the Cochrane Handbook (Section 10.11.4), this technique requires approximately 10 studies per covariate to provide stable estimates, a threshold not met by any of our pooled analyses.^26^ Publication bias was planned to be assessed using funnel plot visualization and Egger's regression test when ≥ 10 studies were available for a specific outcome. All statistical analyses were performed using Review Manager (RevMan) Version 5.4 software.

## Results

### Literature search and characteristics of included studies

**Fig. 1:**
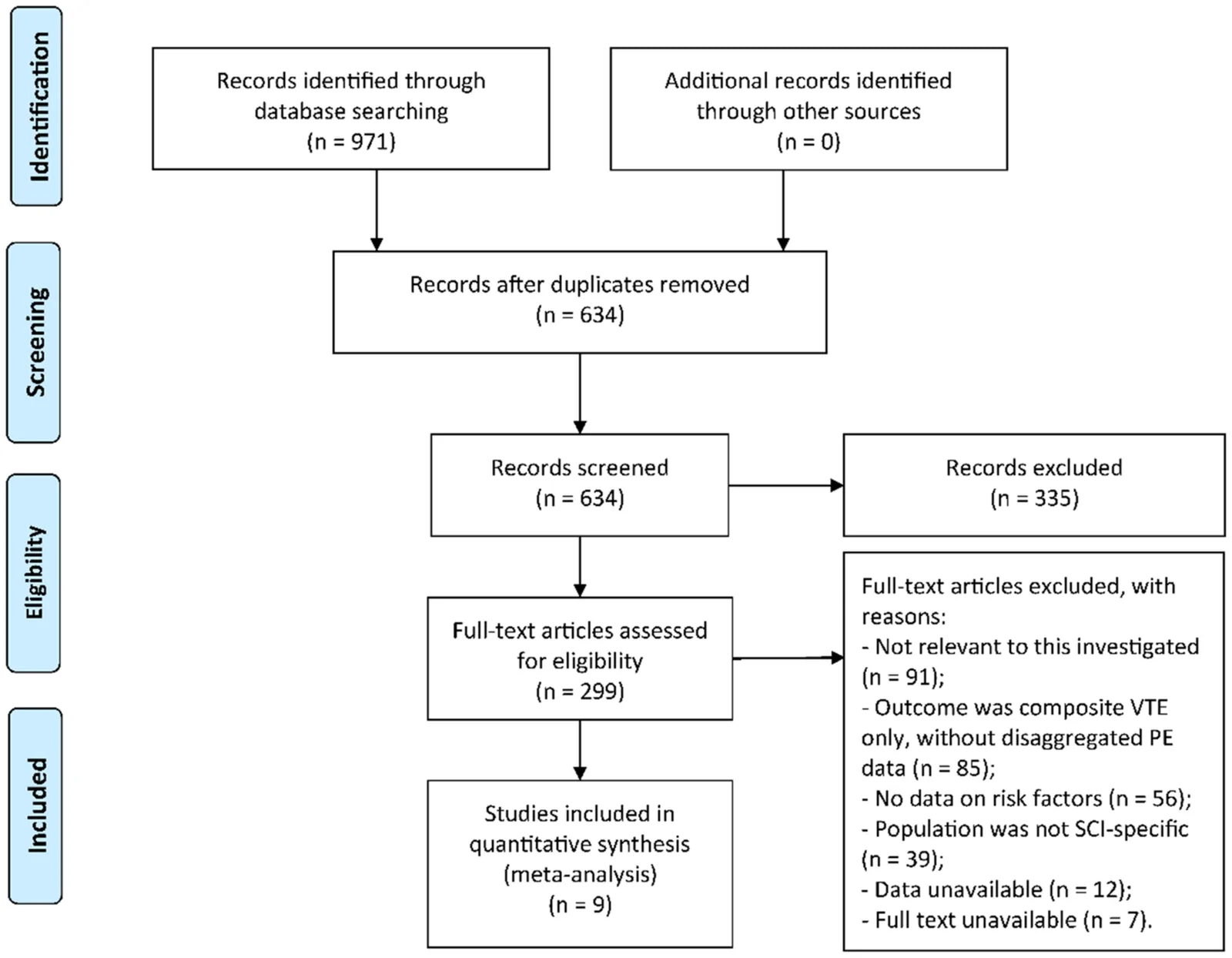
A flowchart of the meta-analysis.

The systematic literature search identified a total of 971 records from electronic databases. After removing 337 duplicates, 634 records were screened based on titles and abstracts. Of these, 335 records were excluded as they did not meet the inclusion criteria, leaving 299 full-text articles for detailed eligibility assessment. Ultimately, nine studies were included in this meta-analysis (Fig. 1).27., 28., 29., 30., 31., 32., 33., 34., 35. The characteristics of the included studies are summarized in Table 1. The studies were published between 2012 and 2024, with sample sizes ranging from 222 to 309,049 participants. The total number of participants across all included studies was 39,4322, of whom 494 developed pulmonary embolisms, yielding an overall incidence rate of 0.125%. The quality assessment revealed that five studies were rated as high quality, four as moderate quality.

**Table 1:** Main characteristics of the included studies.

Author	Year	Country	Sample size	Number of pulmonary embolisms	Age	Sex (Male/ Female)	NOS
Chung	2014	China	309,049	100	49.5 ± 19.9	191,738/117,311	8
Clements	2017	Australia	222	25	44 (25–61)	174/48	6
Cloney	2018	USA	1269	35	59.94±61	645/624	7
Cloney	2020	USA	6869	46	54.9 ± 18.9	3675/3194	7
Kim	2023	Korea	271	17	62.7 ± 15.9	177/94	6
Masuda	2012	Japan	47,743	50	NR	28,348/19,395	7
Ong	2023	Malaysia	375	25	48.63±17.45	251/124	5
Schoenfeld	2013	USA	27,684	113	56.4 ± 15.1	14,221/13,463	7
Li	2024	China	840	83	NR	586/254	6

Age is presented as mean ± standard deviation (years) where reported as a normally distributed continuous variable; where the primary study reported median (IQR), this is indicated explicitly. NR, Not Reported; NOS, Newcastle-Ottawa Scale.

### Meta-analysis of risk factors for pulmonary embolism

**Fig. 2:**
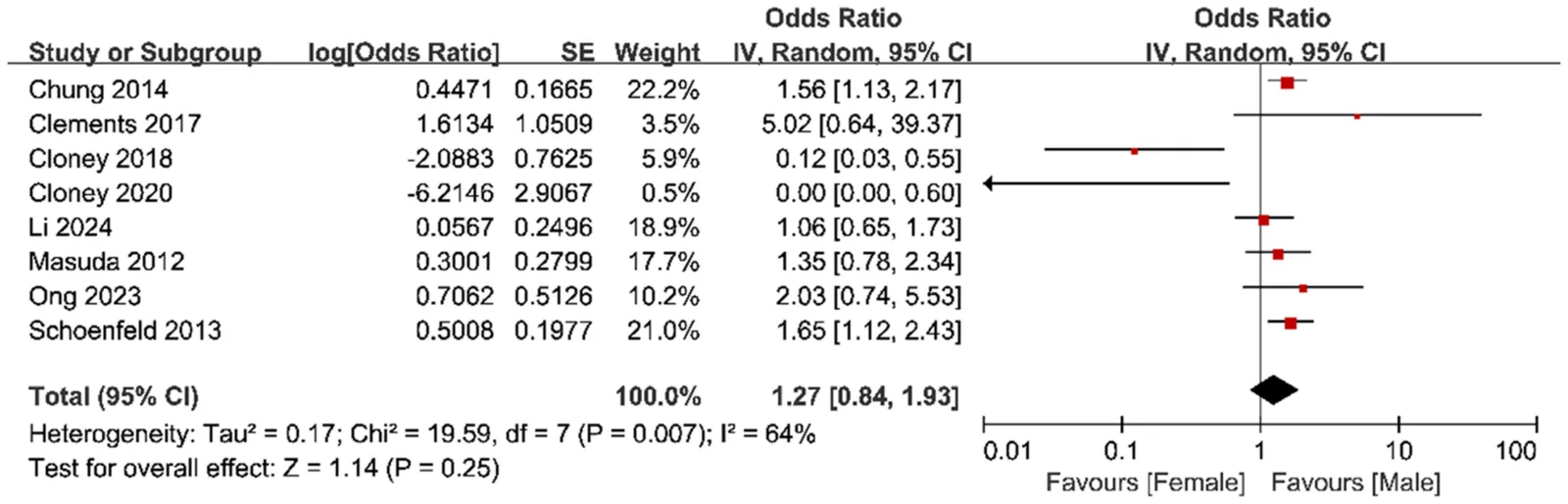
Forest plot of the association between sex and pulmonary embolism risk. Footnote: Pooled estimate from eight studies (n = 394,051). DerSimonian-Laird random-effects model was applied due to substantial heterogeneity (*I*^2^ = 64%, p = 0.007). SE, Standard Error; CI, Confidence Interval.

The meta-analysis of eight studies encompassing 39,4051 patients examined the association between sex and PE risk in SCI patients (Fig. 2, Table 2). Statistical heterogeneity among studies was substantial (*I*^2^ = 64%, p = 0.007), the random effects was therefore utilized. The pooled analysis demonstrated that male sex was not significantly associated with an increased risk of PE compared to female sex (OR = 1.27, 95% CI: 0.84–1.93, p = 0.25).

**Table 2:** Pooled meta-analysis results for risk factors of pulmonary embolism in patients with spinal cord injury.

Risk factor	n	Total n	Statistical model	Pooled OR (95% CI)	*I*^2^	p-value
Sex	8	394,051	DL random	1.27 (0.84–1.93)	64%	0.25
**Age**	3	357,632	MH fixed	**1.80 (1.49–2.17)**	35%	**<0.001**
Body mass index	2	28,953	DL random	1.35 (0.48–3.78)	87%	0.56
**Prolonged operative time**	4	NR	MH fixed	**2.56 (1.84–3.56)**	0%	**<0.001**
Presence of comorbidity	3	317,187	DL random	1.14 (0.33–3.88)	74%	0.84
**Prior history of DVT or PE**	3	NR	MH fixed	**29.10 (14.03–60.37)**	0%	**<0.001**
**Traumatic injury etiology**	5	56,992	DL random	**3.15 (1.37–7.22)**	72%	**0.007**

Statistically significant outcomes (p < 0.05) are highlighted in green and bolded. The fixed-effects (Mantel-Haenszel) model was applied when heterogeneity was low (*I*^2^ ≤ 50%); the random-effects (DerSimonian-Laird) model was applied when heterogeneity was substantial (*I*^2^ > 50%).

N, Number of contributing studies; n, Total participants pooled across contributing studies; OR, Odds Ratio; CI, Confidence Interval; DL, DerSimonian-Laird random-effects model; MH, Mantel-Haenszel fixed-effects model; NR, Not Reported; DVT, Deep Vein Thrombosis; PE, Pulmonary Embolism.

**Fig. 3:**
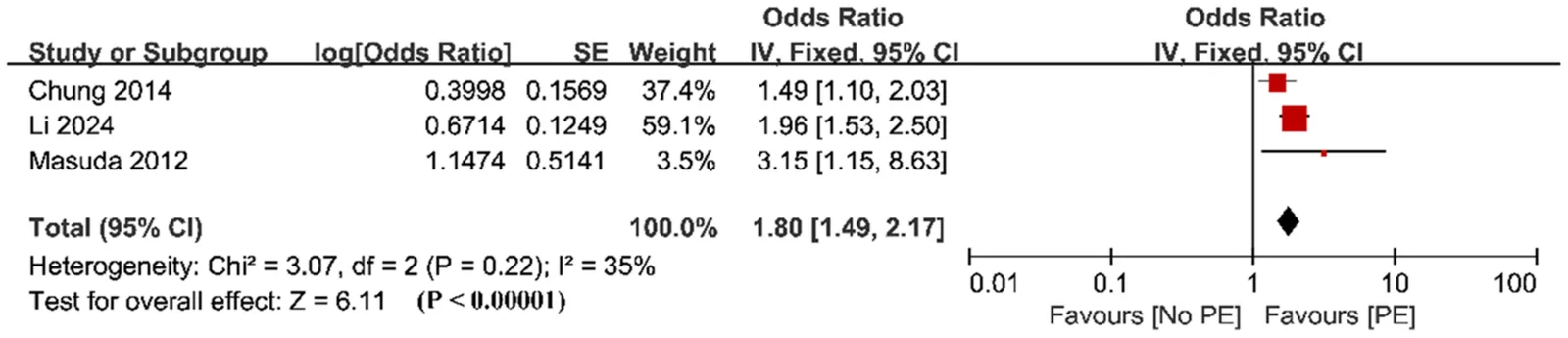
Forest plot of the association between age and pulmonary embolism risk. Footnote: Pooled estimate from three studies (n = 357,632). Mantel-Haenszel (MH) fixed-effect was applied due to low heterogeneity (*I*^2^ = 35%, p = 0.22). Bold values statistical significance (p < 0.05). SE, Standard Error; CI, Confidence Interval; PE Pulmonary Embolism.

Three studies involving 35,7632 participants reported age data for meta-analysis (Fig. 3, Table 2). The heterogeneity analysis revealed low heterogeneity (*I*^2^ = 35%, p = 0.22), the fixed-effect was therefore utilized. The pooled results indicated that patients who developed PE were significantly older compared to those without PE (OR = 1.80, 95% CI: 1.49–2.17, p < 0.001). This estimate is based on a small number of studies and should be interpreted as hypothesis-generating.

**Fig. 4:**
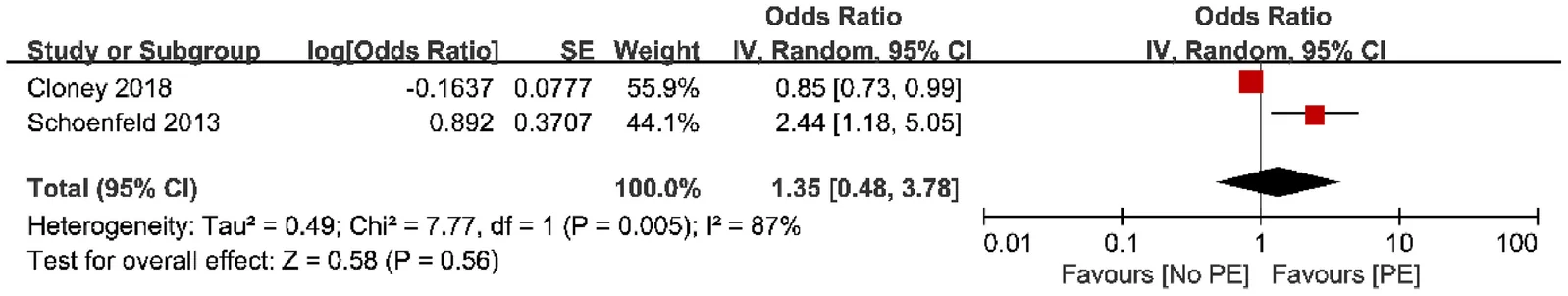
Forest plot of the association between body mass index and pulmonary embolism risk. Footnote: Pooled estimate from two studies (n = 28,953). DerSimonian-Laird random-effects model was applied due to substantial heterogeneity (*I*^2^ = 87%, p = 0.005). SE, Standard Error; CI, Confidence Interval; PE Pulmonary Embolism.

The relationship between BMI and PE risk was evaluated in two studies with a total of 28,953 patients (Fig. 4, Table 2). Heterogeneity testing indicated substantial heterogeneity across studies (*I*^2^ = 87%, p = 0.005), the random-effects was therefore utilized. The pooled analysis showed that patients with PE had similar BMI values compared to those without PE (OR = 1.35, 95% CI: 0.48–3.78, p = 0.56). Given that only two studies contributed, this analysis is exploratory; the wide confidence interval is compatible with both clinically important and clinically unimportant associations.

**Fig. 5:**
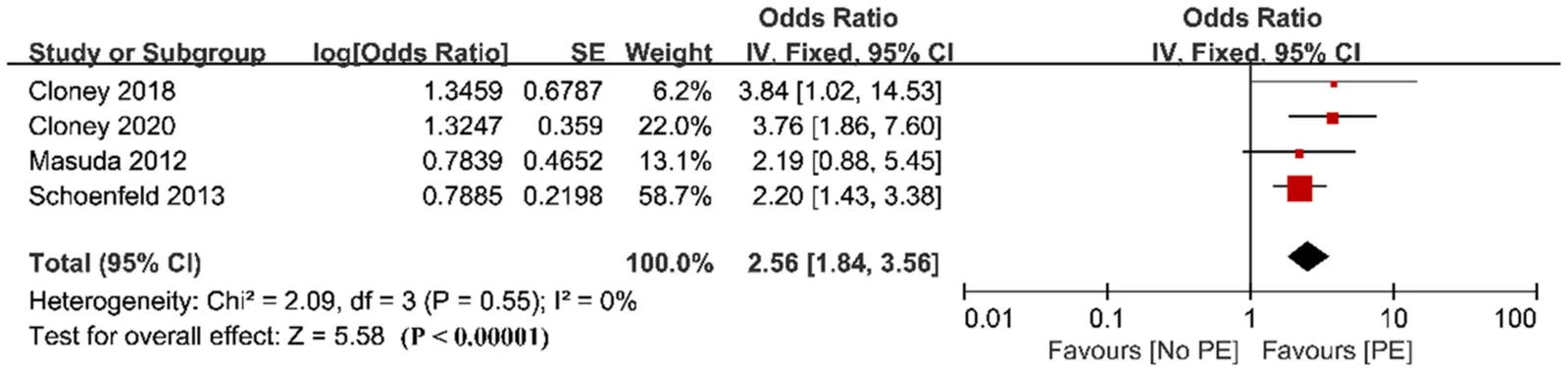
Forest plot of the association between prolonged operative time and pulmonary embolism risk. Footnote: Pooled estimate from four studies (n = 83,565). Mantel-Haenszel (MH) fixed-effect model was applied because no heterogeneity was detected (*I*^2^ = 0%, p = 0.55). Bold values statistical significance (p < 0.05). SE, Standard Error; CI, Confidence Interval; PE Pulmonary Embolism.

Four studies reporting operative time data were pooled for meta-analysis (Fig. 5, Table 2). There was no heterogeneity (*I*^2^ = 0%) among included studies, the fixed-effect was therefore utilized. The results demonstrated that longer operative time was significantly associated with PE risk (OR = 2.56, 95% CI: 1.84–3.56, p < 0.001).

**Fig. 6:**
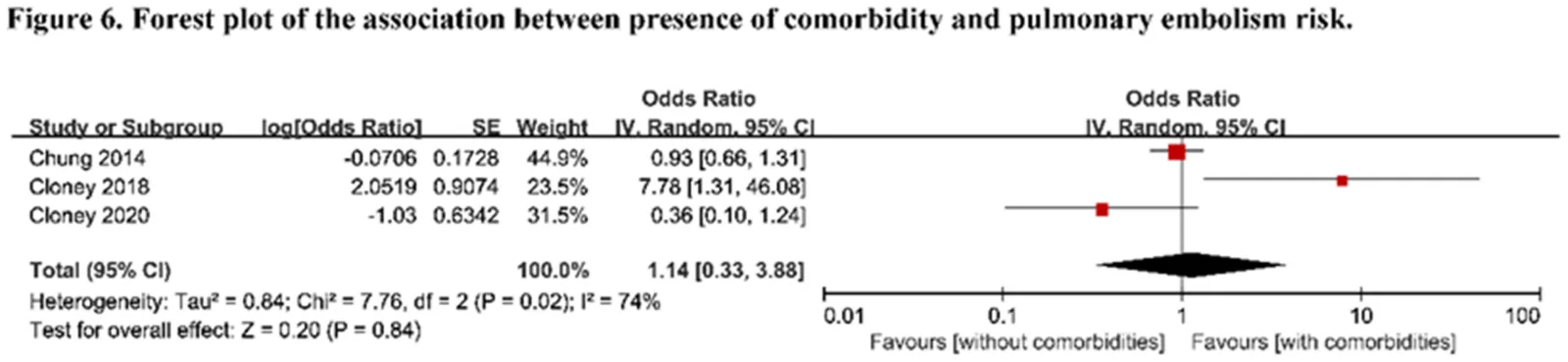
Forest plot of the association between presence of comorbidity and pulmonary embolism risk. Footnote: Pooled estimate from three studies (n = 317,187). DerSimonian-Laird random-effects model was applied due to substantial heterogeneity (*I*^2^ = 74%, p = 0.02). SE, Standard Error; CI, Confidence Interval.

The pooled analysis of three studies with 317,187 patients examined the impact of comorbidities on PE risk (Fig. 6, Table 2). Statistical heterogeneity among studies was substantial (*I*^2^ = 74%, p = 0.02), the random-effects was therefore utilized. Patients with comorbidities had similar odds of developing PE compared to those without comorbidities (OR = 1.14, 95% CI: 0.33–3.88, p = 0.84).

**Fig. 7:**
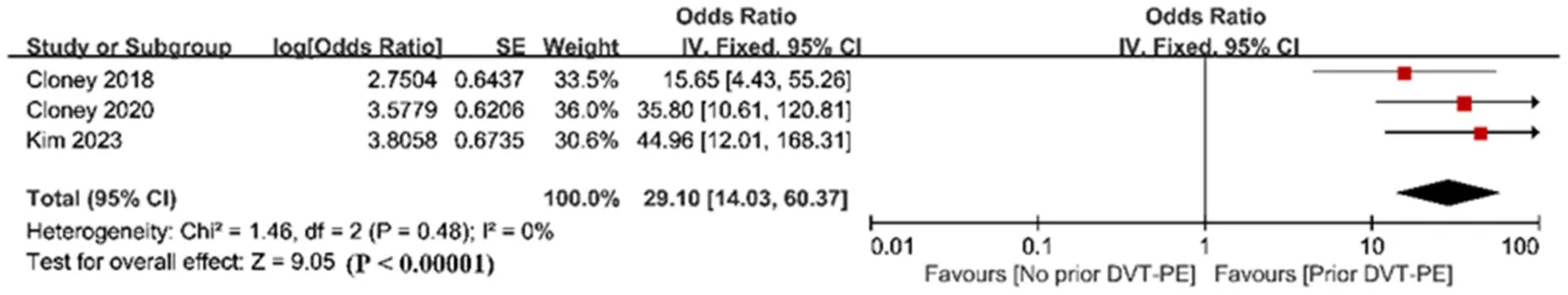
Forest plot of the association between history of deep vein thrombosis or pulmonary embolism and subsequent PE risk. Footnote: Pooled estimate from three studies (n = 8409). Mantel-Haenszel (MH) fixed-effect model was applied because no heterogeneity was detected (*I*^2^ = 0%, p = 0.48). Bold values statistical significance (p < 0.05). SE, Standard Error; CI, Confidence Interval; PE Pulmonary Embolism; DVT, Deep Vein Thrombosis.

Three studies reporting prior DVT or PE history were included in the meta-analysis (Fig. 7, Table 2). There was no heterogeneity (*I*^2^ = 0%) among included studies, the fixed-effect was therefore utilized. The pooled results revealed that a history of DVT or PE was strongly associated with recurrent PE risk in SCI patients (OR = 29.10, 95% CI: 14.03–60.37, p < 0.001). Although directionally consistent across all three contributing studies, the wide 95% CI reflects this limited evidence base; the absolute magnitude of effect should therefore be interpreted with corresponding caution.

**Fig. 8:**
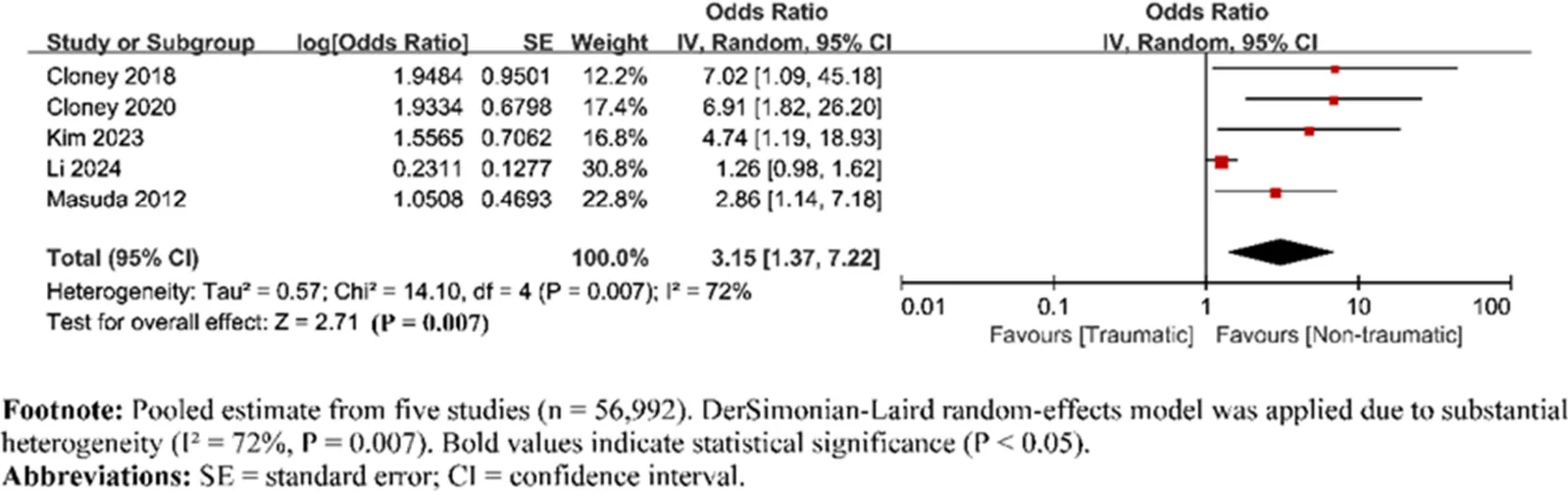
Forest plot of the association between traumatic etiology of spinal cord injury and pulmonary embolism risk. Footnote: Pooled estimate from five studies (n = 56,992). DerSimonian-Laird random-effects model was applied due to substantial heterogeneity (*I*^2^ = 72%, p = 0.007). SE, Standard Error; CI, Confidence Interval.

The association between traumatic injury etiology and PE risk was assessed in five studies involving 56,992 participants (Fig. 8, Table 2). Statistical heterogeneity was substantial (*I*^2^ = 72%, p = 0.007), the random-effects was therefore utilized. The meta-analysis showed that traumatic causes of SCI were significantly associated with PE development compared to non-traumatic causes (OR = 3.15, 95% CI: 1.37–7.22, p = 0.007).

### Exploration of heterogeneity

Substantial-to-considerable heterogeneity was observed for the analyses of sex (*I*^2^ = 64%), BMI (*I*^2^ = 87%), comorbidities (*I*^2^ = 74%), and traumatic etiology (*I*^2^ = 72%). Several potential sources were identified. First, the included studies originated from heterogeneous healthcare systems (United States, China, Europe) with markedly different VTE prophylaxis protocols and surveillance intensities, which directly affect the baseline PE incidence against which risk-factor effects are measured. Second, PE ascertainment varied across studies, ranging from systematic Computed Tomography Pulmonary Angiography (CTPA)-based screening to clinically-driven imaging only, predisposing to differential outcome detection. Third, the operational definition of “comorbidity” was inconsistent (Charlson Comorbidity Index, individual disease counts, or composite scores). Fourth, the studies spanned 2012–2024, during which prophylaxis standards evolved substantially. These differences are intrinsic to the available primary literature and could not be fully resolved by re-analysis.

### Sensitivity analysis

Sensitivity analyses were conducted by sequentially excluding individual studies to assess the robustness of the pooled estimates. The results remained stable after removing any single study, indicating robust findings (Supplementary Table S2).

### Publication bias

Due to the limited number of included studies (n = 9), formal assessment of publication bias using funnel plot asymmetry tests was not performed.

## Discussion

This meta-analysis synthesizes data from 394,322 SCI patients across nine studies to provide quantification of PE risk factors in this vulnerable population. Our findings establish four positive risk-factor associations: older age (OR = 1.80, 95% CI: 1.49–2.17), prolonged operative time (OR = 2.56, 95% CI: 1.84–3.56), prior thromboembolic history (OR = 29.10, 95% CI: 14.03–60.37), and traumatic injury etiology (OR = 3.15, 95% CI: 1.37–7.22). No statistically significant associations were detected for sex, BMI, or composite comorbidity. The overall PE incidence of 0.125% observed in our pooled analysis is substantially lower than historically reported hospitalization-phase rates of 0.6%–5%.^6^ consistent with the introduction of contemporary prophylactic protocols. These findings may inform the future development of SCI-specific risk-stratification tools.

The four positive risk factors identified converge mechanistically through amplification of Virchow's triad ‒ venous stasis, endothelial injury, and hypercoagulability ‒ which is fundamentally disrupted in SCI. Neurogenic paralysis eliminates skeletal-muscle pump function while autonomic dysregulation impairs venous compliance, producing profound lower-extremity stasis.^36^ Impaired fibrinolysis driven by PAI-1 elevation compromises endogenous clot resolution and sustains a prothrombotic state for weeks-to-months post-injury. The 1.8-fold increased risk associated with older age is consistent with age-related baseline hypercoagulability, including endothelial senescence with diminished nitric-oxide bioavailability, upregulation of von Willebrand factor and tissue factor, and declining fibrinolytic capacity.^14^ The very large effect magnitude for prior DVT/PE history (OR = 29.10) is biologically plausible given that residual venous obstruction, valvular incompetence, and persistent endothelial dysfunction collectively produce a sustained recurrence-prone substrate.^9^ Prolonged operative time (OR = 2.56) reflects multiple thrombogenic pathways including procoagulant tissue factor release, IL-6 and TNF-α-mediated endothelial activation, and consumptive depletion of natural anticoagulants.^37^ Traumatic injury mechanisms (OR = 3.15) compound risk through direct endothelial disruption, concurrent long-bone and pelvic injuries that multiply tissue factor release, and inflammatory phenomena promoting immunothrombosis.^38^

Existing generic VTE risk-assessment tools (Caprini, Padua, Wells, Geneva, IMPROVE) demonstrate limited discrimination in SCI populations because they universally categorize all SCI patients as high-risk without enabling within-group differentiation.^18^^,^^19^^,^^39^ Recent validation work demonstrates that integration of SCI-specific variables modestly improves discriminative performance (Area Under the Curve [AUC] 0.751 to 0.808).^39^ Our four positive risk factors are conceptually compatible with the structure of such an SCI-tailored tool, but prospective derivation, internal calibration, and external validation across diverse populations are necessary before any specific score can be recommended for clinical use.

The optimal pharmacological agent, dose, initiation timing, duration, and surveillance strategy for VTE prophylaxis in SCI remain matters of ongoing investigation and guideline divergence. Low-Molecular-Weight Heparin (LMWH) is supported as the first-line prophylactic agent by multiple guidelines.^36^^,^^40^ and the Spinal Cord Injury Thromboprophylaxis Investigators (SCITI) trial demonstrated lower PE incidence with enoxaparin monotherapy compared with Low-Dose Unfractionated Heparin (LDUH) plus intermittent pneumatic compression (5.2%vs. 18.4%, p = 0.03).^41^ Early initiation of pharmacologic VTE prophylaxis after SCI is supported by observational data, including a prospective Transforming Research and Clinical Knowledge (TRACK)-SCI registry study.^42^ which demonstrated a low rate of spine surgery–related bleeding when LMWH was initiated within 24-hours of injury or surgery, and a Consortium of Leaders in the Study of Traumatic Thromboembolism (CLOTT) secondary analysis in SCI by Godat et al.^43^ which found that initiation within 48-hours was independently associated with lower odds of VTE compared with later initiation. However, our meta-analysis does not directly address prophylactic regimens, and we therefore refrain from making specific recommendations regarding agent selection, dose, or duration.

Our findings regarding sex, BMI, and composite comorbidity warrant cautious interpretation. The absence of statistically significant associations should not be interpreted as evidence of no effect, particularly for BMI (only 2 studies, n = 28,953) where the wide 95% CI (0.48–3.78) is compatible with both clinically important and unimportant effects. Other meta-analyses with different inclusion criteria have reported small associations for male sex and certain individual comorbidities^44^ The pragmatic interpretation is that these factors, in isolation, do not appear to dominate PE risk in the available evidence base, but multiple concurrent comorbidities or extreme phenotypes (e.g., morbid obesity) may still warrant individualized clinical attention.

Diagnostic challenges in SCI include sensory deficits masking classic symptoms, atypical PE presentations, and non-specific baseline bilateral lower-extremity edema.^45^ A recent trauma meta-analysis reported an 18% PE prevalence among CTPA examinations (95% CI 13–24%).^46^ supporting a lowered imaging threshold in patients with lower-extremity or spine fractures. Surveillance strategies remain controversial: A Virginia Commonwealth University trauma-ICU surveillance study in a general trauma population showed that twice-weekly lower-extremity duplex ultrasonography increased DVT detection and was associated with a reduction in PE incidence from 1.5% to 0.7% (p < 0.05), with an estimated incremental cost of $29,102 per QALY gained.^47^ However, because this was not an SCI-specific cohort, the generalisability of these findings to patients with SCI remains uncertain, and prospective SCI-specific evaluation is warranted.

Our meta-analysis has several limitations. First, several pooled estimates were derived from a small number of primary studies (2 studies for BMI; 3 studies each for age, prior DVT/PE, and comorbidities). For these analyses, the precision of point estimates is limited, and the absence of statistical significance must not be interpreted as evidence of no effect; findings derived from ≤ 3 studies should be regarded as hypothesis-generating and require confirmation by future large-scale, prospective, multivariable-adjusted investigations. Second, the predominance of observational study designs limits causal inference, with potential residual confounding from differences in prophylaxis protocols and injury severity. Third, study heterogeneity spanning diverse geographic regions, healthcare systems, and prophylaxis standards introduced baseline PE incidence variability. Fourth, follow-up durations concentrated within 90-days potentially underestimate long-term VTE risk. Fifth, diagnostic-criteria heterogeneity (symptomatic vs. asymptomatic VTE, varying imaging modalities) affects between-study comparability. Sixth, publication bias risk cannot be excluded given the limited number of available studies (n = 9); formal funnel plot or Egger's test assessment was precluded by the small number of studies per outcome.

Future research priorities include: 1) Large multicentre prospective studies of PE-specific (not composite VTE) outcomes in SCI with standardized multivariable risk-factor reporting, to permit improved pooled estimates and meta-regression; 2) Prospective derivation and external validation of SCI-specific risk-prediction scores; 3) Trials of optimal LMWH dosing strategies and prophylaxis duration with PE-specific endpoints; and 4) SCI-specific evaluations of direct oral anticoagulants for both acute and rehabilitation-phase prophylaxis.

## Conclusion

This meta-analysis establishes an evidence base associating four risk factors ‒ older age, prolonged operative time, prior thromboembolic history, and traumatic injury etiology ‒ with increased PE risk in SCI patients, with effect magnitudes ranging from 1.8-fold to 29-fold. These factors converge mechanistically through amplification of Virchow's triad and may inform the future development of SCI-specific risk-stratification frameworks following prospective validation. The absence of significant associations for sex, BMI, and routine comorbidity should not be over-interpreted as definitive null findings given the limited study count for several individual analyses. Translation of our findings into specific clinical decision rules ‒ including any specific prophylactic regimen, dose, or duration recommendation ‒ requires prospective derivation and external validation, which constitutes a critical priority for future research.

## Ethical statement

As this is a secondary literature-based study, ethic approval is not necessary.

## Funding

No funding was received for this work.

## Data availability

The datasets used and/or analyzed during the current study are available from the corresponding author on reasonable request.

## Conflicts of interest

The authors declare no have conflicts of interest.

## Appendix Supplementary materials

Image, application 1
