# Supplementary material for: Analysis of risk factors associated with pulmonary embolism in patients with spinal cord injury: A meta-analysis
Source: Clinics (Sao Paulo). 2026 Sep 6;81:101095. doi: 10.1016/j.clinsp.2026.101095 (PMC13579303; doi:10.1016/j.clinsp.2026.101095)
Supplement: Supplementary file 1 [file mmc1.docx]

**CLINICS-D-26-00425_Supplementary Material**

**Supplementary Table S1** Leave-one-out sensitivity analysis of pooled estimates.

| **Study removed** | **Pooled OR** | **95% CI** | ***I*^2^** |
| --- | --- | --- | --- |
| **Outcome 1: Sex ‒ Base-case pooled estimate: OR = 1.27 (0.84–1.93); *I*^2^ = 64%** | | | |
| Excluding Chung 2014 | 1.16 | 0.66–2.03 | 68% |
| Excluding Clements 2017 | 1.21 | 0.80–1.84 | 67% |
| Excluding Cloney 2018 | 1.39 | 0.98–1.75 | 65% |
| Excluding Cloney 2020 | 1.33 | 0.93–1.92 | 59% |
| Excluding Li 2024 | 1.3 | 0.79–2.15 | 67% |
| Excluding Masuda 2012 | 1.23 | 0.74–2.04 | 69% |
| Excluding Ong 2023 | 1.2 | 0.76–1.88 | 69% |
| Excluding Schoenfeld 2013 | 1.15 | 0.67–1.98 | 68% |
| **Outcome 2: Operative time ‒ Base-case pooled estimate: OR = 2.56 (1.84–3.56); *I*^2^ = 0%** | | | |
| Excluding Cloney 2018 | 2.49 | 1.77–3.51 | 0% |
| Excluding Cloney 2020 | 2.3 | 1.58–3.34 | 0% |
| Excluding Masuda 2012 | 2.62 | 1.84–3.74 | 0% |
| Excluding Schoenfeld 2013 | 3.18 | 1.90–5.31 | 0% |
| **Outcome 3: Traumatic injury etiology ‒ Base-case pooled estimate: OR = 3.15 (1.37–7.22); *I*^2^ = 72%** | | | |
| Excluding Cloney 2018 | 2.79 | 1.18–6.62 | 74% |
| Excluding Cloney 2020 | 2.55 | 1.12–5.77 | 66% |
| Excluding Kim 2023 | 2.91 | 1.16–7.29 | 74% |
| Excluding Li 2024 | 4.26 | 2.28–7.97 | 63% |
| Excluding Masuda 2012 | 3.49 | 1.14–10.65 | 75% |

OR, Odds Ratio; CI, Confidence Interval.
